# A Study of Skin Cancer Knowledge, Attitudes, and Preventive Practices Among Healthcare Professionals and the General Population in Pakistan: Insights for Healthcare Interventions and Policy Development

**DOI:** 10.1155/jskc/3035274

**Published:** 2025-03-20

**Authors:** Adeel Aslam, Shazia Jamshed, Asma Ghulam Mustafa, Suresh Shanmugham, Abubakar Wazir, Ali hussnain, Maha Amjad, Hafiz Muhammad Bilal, Zahra Moslemifard Khaledi

**Affiliations:** ^1^Faculty of Pharmacy and Biomedical Sciences, Mahsa University, Jenjarom, Selangor, Malaysia; ^2^Department of Pharmacy Practice, International Medical University, Kuala Lumpur, Malaysia; ^3^Department of Pharmacy, University of Lahore, Punjab, Lahore, Pakistan

**Keywords:** general population knowledge, healthcare professionals' knowledge, skin cancer attitude, skin cancer Pakistan, skin cancer practices

## Abstract

**Background:** Skin cancer is a major global health issue that can be life-threatening. The current study explores the knowledge, attitudes, and practices of healthcare professionals and the general population regarding skin cancer.

**Method:** A cross-sectional study was conducted between January and June 2023 in Lahore, the second-largest city in Pakistan. A total of 407 respondents from the general population and 230 healthcare professionals were recruited using a convenient and snowball sampling technique, respectively. Data were collected using questionnaires and statistical analysis, including chi-square tests, and bivariate logistic regression was performed using SPSS Version 20.

**Result:** In the overall population, 46.4% identified as male, 45.0% as female, and 8.6% chose not to disclose their gender. Significantly differing levels of skin cancer knowledge were observed between genders, with males reporting a higher knowledge (36.4%, crude odds ratio = 1.413, *p* < 0.001) compared to females (32.9%). In addition, females displayed a more positive attitude (crude odds ratio = 1.874, *p* < 0.001) and practice (crude odds ratio = 1.401, *p* < 0.05) toward skin cancer prevention. Furthermore, younger age groups exhibited greater knowledge, attitudes, and practices regarding skin cancer. Education and employment status also have a direct influence on skin cancer knowledge and practices. Moreover, in the current study, healthcare professionals comprised 61.3% physicians, 16.5% nurses, and 22.2% pharmacists. Among these, 37.3% of physicians, 11.3% of nurses, and 12.6% of pharmacists demonstrated the ability to identify common signs and symptoms of skin cancer in a patient (*p* < 0.001). Furthermore, 44% of physicians regarded regular skin cancer screenings as “very important” (*p* < 0.01). In addition, 27.4% of physicians and 8.7% of pharmacists exhibited a positive attitude toward regular screening of at-risk patients (*p* < 0.05).

**Conclusion:** The current study highlights gender and age disparities in skin cancer knowledge and prevention among the general population, emphasizing the need for targeted public health interventions to enhance knowledge and proactive practices. In addition, among healthcare professionals, it reveals variations in knowledge levels, emphasizing the importance of tailored education initiatives to promote consistent skin cancer prevention and management practices within the healthcare community.

## 1. Introduction

Skin cancer is a major global health issue that can be life-threatening [1]. It starts in the cells of the skin and is one of the most common types of cancer worldwide [2]. Skin cancer, ranks as the fifth most common cancer globally, making up about 5.8% of all cancer cases, with melanoma accounting for 1.6% [3, 4]. It carries a mortality rate of 0.6%–0.7% [5]. Interestingly, in Pakistan, where there is no national-level system to track cancer cases, regional cancer registries, such as the Punjab Cancer Registry (PCR), have raised concerns about the increasing cases of skin cancer [6]. This is in sharp contrast to the Globocan 2018 report, which ranked nonmelanoma skin cancers (NMSCs) 18th and melanoma 32nd among the most common cancers in the country [7]. Excessive exposure to ultraviolet (UV) radiation can be considered the leading cause, especially in recent years, due to the reduction in the ozone layer [8]. It has shown significant differences between UVA and UVB radiation, such as ionizing radiation and the ability to penetrate the skin [9]. UVB is the leading cause of cutaneous neoplasm, even though it only represents 5% of solar radiation [9, 10]. This is because UVB radiation causes the appearance of point mutations in target genes [11]. In Pakistan, a country characterized by abundant sunlight and cultural diversity, skin cancer has emerged as a growing health concern that demands immediate attention [12]. The rise in skin cancer cases can also be attributed to shortcomings in diagnosis within both primary and secondary healthcare, along with unnecessary exposure to environmental factors by the population [13, 14]. This exposure often occurs because of a lack of knowledge about skin cancer [15]. This disease does not discriminate, affecting people of all ages and ethnic backgrounds, making prevention and early detection critical in mitigating its impact [16]. Healthcare professionals, including physicians, nurses, and allied healthcare workers, wield substantial influence by disseminating accurate information, offering preventive guidance, and facilitating timely diagnosis and treatment [17]. Healthcare professionals emphasize periodic visual skin examinations, dermoscopy, and biopsies as essential tools for early detection of skin cancer. In addition, they stress the importance of patient education on self-examinations using the asymmetry, border irregularity, color variation, diameter > 6 mm, and evolution (ABCDE) rule to empower early diagnosis [18]. However, it is important to note that the extent of their knowledge, attitudes, and practices regarding skin cancer in Pakistan remains unexplored [19]. Nevertheless, the fight against skin cancer goes beyond the healthcare community. Raising knowledge about the risks and encouraging preventive measures among the general population are equally important [20]. Indeed, factors such as social beliefs, educational disparities, and access to healthcare resources play a significant role in shaping the public's understanding and response to this challenging issue [21]. There was limited focus on skin cancer prevention and awareness initiatives, particularly in low- and middle-income countries (LMICs), where resources and public health priorities differ markedly from high-income settings [22]. A comprehensive assessment of the Pakistani population's and healthcare professional's knowledge, attitudes, and practices is essential for designing targeted public health interventions. Despite the importance of understanding both healthcare professionals' and the general public's perspectives on skin cancer, research in Pakistan has been limited. Most existing studies have primarily focused on statistics and prevalence, overlooking the influence of culture and society on people's experiences with this disease [6, 7]. Therefore, the current research aims to bridge this gap by exploring the knowledge, attitudes, and practices of healthcare professionals and the general population in Pakistan regarding skin cancer.

## 2. Materials and Methods

### 2.1. Study Design and Study Setting

A cross-sectional study was conducted between January and June 2023 to assess the knowledge, attitudes, and practices regarding skin cancer among healthcare professionals and the general population in Lahore, Pakistan's second-largest city [23].

### 2.2. Study Population and Sample Size

Participants in the current study include the general population and healthcare professionals (physicians, nurses, and pharmacists) who are 18 and older. The study's objectives were explained to potential participants, who voluntarily agreed to take part and signed an informed consent form. The consent process took place in a private setting, allowing participants to ask questions and ensuring they fully understood their involvement in the study. To address potential risks, the study design minimized participant exposure to sensitive questions, and any personal information collected was kept confidential and anonymized. Participation was entirely voluntary, and no incentives were offered. Furthermore, to manage any possible conflicts of interest, all members of the research team were required to disclose any affiliations or interests related to the study, and participants were informed that the study was conducted independently without external funding that could bias the results. Participants who did not meet the abovementioned inclusion criteria were excluded from our study. Furthermore, individuals who declined to provide informed consent and those who submitted incomplete questionnaires were excluded from the study. The sample size was determined with a 95% confidence level using Rasoft software, which indicated a minimum requirement of 385 subjects from each population. The current study's primary focus was collecting data from the general population, with a target of 450 respondents. Of these, 407 questionnaires were completely filled and included in the study. In addition, we aimed to survey 450 respondents from healthcare professionals, but only 300 consented, and 230 questionnaires were fully completed due to the hospital's approval for a limited 6-month data collection period, all of which were included in our study. This response rate reflects the common challenges in healthcare settings, where busy schedules and patient care duties often make it difficult for healthcare professionals to participate. Consequently, the final sample comprised 407 respondents from the general population and 230 from healthcare professionals.

### 2.3. Study Instrument

The study data were gathered using questionnaires (Supporting 1) developed based on a thorough literature review [11, 24–26] and expert consultations to ensure cultural and contextual relevance. Questionnaires were customized separately for healthcare professionals and the general population. Each questionnaire underwent a pilot test with 30 individuals (15 from the general population and 15 healthcare professionals) to assess the items' clarity, relevance, and comprehension. Feedback from the pilot phase was used to refine question wording, improve question order, and ensure the questionnaires were culturally appropriate. The final questionnaires exhibited satisfactory internal consistency, as indicated by their respective Cronbach's alpha values. For Questionnaire 1 (general population), the Cronbach's alpha was 0.725, while for Questionnaire 2 (healthcare professionals), it was 0.675. Construct validity was also assessed, with Questionnaire 1 achieving a score of 0.98 and Questionnaire 2 a score of 0.85, confirming the reliability and consistency of the results. The content and face validity of the final version of Questionnaire 1 were evaluated by a panel of eight experts in pharmacy practice, while Questionnaire 2 was evaluated by a panel of eight physicians. The content validity index (CVI) was 0.89 for Questionnaire 1 and 0.87 for Questionnaire 2, indicating strong validity for both questionnaires.

The final version of the questionnaire comprised five sections, covering sociodemographic characteristics, knowledge, attitudes, and practices related to skin cancer, as well as the sources from which participants acquired their knowledge. All sections contained close-ended questions. In Questionnaire 1, the demographic section contains four items, while the knowledge, attitude, and practices section each comprises five items, and the information source section contains two items. In Questionnaire 2, the demographic section also includes four items. In contrast, the knowledge, attitude, and practices section consists of three items each, and the information source section contains only one item.

### 2.4. Sampling Technique and Data Collection

Using a convenience sampling technique, data from the general population were collected in public settings, such as areas outside large hospitals and shopping malls. This technique was selected due to its practicality and accessibility in targeting diverse participants within the available timeframe. On the other hand, data from healthcare professionals were collected using a snowball sampling technique at the largest government tertiary care hospital in Lahore after obtaining their approval. This technique was selected to reach more healthcare professionals through their professional connections.

### 2.5. Data Analysis

The data analysis process was carried out by using the Statistical Package for Social Sciences (SPSS) Version 20. Descriptive statistics, such as frequency and percentage, were used to analyze the characteristics of the study population. Inferential statistics were employed in the form of a chi-square test to assess the significance of the association between participants' gender and their knowledge about skin cancer. Bivariate comparisons were examined using chi-square statistics. Statistical significance was defined as a *p* value less than 0.05. Logistic regression analysis was employed to compute both crude and adjusted odds ratios (ORsa), along with their respective 95% CI. Initially, 1 point was assigned for every correct answer, while 0 points were assigned for each incorrect response; then, all correct answers were individually introduced into multinomial logistic regression models to assess their association with sociodemographic characteristics. Subsequently, all predictors were included in a multivariable logistic regression analysis to obtain ORsa. OR examines the relationship between independent and dependent variables without considering other factors. While ORa considers multiple variables simultaneously, assessing the relationship between the exposure and outcome while adjusting for confounding variables could influence this relationship.

### 2.6. Ethical Considerations

The University of Lahore granted ethical approval for the study under IREC-2023-13H, and the research was conducted strictly according to the ethical standards set forth by the institutional research committee. This study is in line with the Declaration of Helsinki, and our university research committee supervised us throughout the study.

## 3. Result

### 3.1. General Population

#### 3.1.1. Demographic Characteristics

Table 1 presents a demographic breakdown of the study participants, shedding light on their gender, age, education levels, and occupations. Among the respondents, 46.4% were male, while 45.0% were female, and 8.6% preferred not to disclose their gender. Regarding age, the majority fell into the 25–34 age group (36.1%), followed by the 18–24 group (35.1%), with smaller proportions in the older age categories. Regarding education, the highest percentage held a bachelor's degree (45.9%), while 23.8% had a master's degree or higher. The study encompassed a diverse group, with 26.0% being employed full-time, 13.5% employed part-time, 14.0% self-employed, 26.8% students, 15.5% unemployed, and 4.2% retired. This demographic distribution provides a comprehensive overview of the study's participant characteristics.

#### 3.1.2. Knowledge of Skin Cancer

Table 2 presents a comprehensive overview of the general population's responses related to skin cancer knowledge and knowledge segmented by gender. Among the key findings, a significant gender difference was observed in the knowledge of skin cancer, with 36.4% of males and 32.9% of females reporting prior knowledge. While there is no statistically significant difference in knowledge levels, a noteworthy trend is observed: 8.8% of males and 6.4% of females consider themselves “very knowledgeable” about skin cancer. Furthermore, the table delves into respondents' understanding of preventive measures and risk factors, indicating that 16.7% of males and 18.2% of females believe wearing sunscreen can prevent skin cancer, and 20.9% of males and 16.2% of females recognize excessive sun exposure as a common risk factor. Among males, 20.6% believe that unusual growth or lumps on the skin are major signs or symptoms of skin cancer, while among females, this belief is held by 17.7%.

#### 3.1.3. Attitude Toward Skin Cancer

Table 3 presents a comprehensive overview of the general population's attitude and behaviors regarding skin cancer knowledge and prevention, stratified by gender. Notably, a majority of both male and female participants believe that skin cancer is a significant health concern (40.3% male and 36.4% female) and are concerned about its potential risks (31.0% male and 30.5% female). Significantly, women tend to be more proactive in preventive measures, with higher percentages practicing sun protection behaviors such as wearing protective clothing (13.0% female and 17.4% male) and seeking shade during peak sun hours (9.3% female, 10.1% male). The data also underscored the importance of regular skin checks by a dermatologist (28.7% male and 21.1% female). Notably, gender differences play a substantial role in these attitudes.

#### 3.1.4. Practices of Skin Cancer Prevention and Management

Table 4 presents a detailed analysis of general population practices and behaviors toward skin cancer knowledge and prevention, categorized by gender. Notably, substantial proportions of respondents, especially males, have not undergone a skin cancer screening or examination (27.0% males and 30.5% females) and have not performed self-examinations for skin cancer signs (25.3% males and 30.5% females). However, a significant gender difference arises in the frequency of sun protection usage, with a higher percentage of females (16.0%) always using sun protection compared to males (11.5%). Moreover, the data indicate that upon noticing suspicious skin changes, most individuals from both genders prefer to schedule an appointment with a healthcare professional (28.3% males and 24.1% females). This underscores the crucial role of timely medical attention in addressing potential skin-related concerns.

#### 3.1.5. Regression Analysis

In Table 5, odds ratios (ORs) and ORsa with their corresponding 95% CI are presented to assess the association between gender, age, education, and occupation with knowledge, attitude, and practices related to skin cancer. Statistically significant associations are denoted by asterisks (⁣^∗^*p* < 0.05; ⁣^∗∗^*p* < 0.01; ⁣^∗∗∗^*p* < 0.001). Both males and females significantly associate various aspects of knowledge, attitudes, and practices. Males exhibit higher odds of having positive knowledge (OR = 1.413, *p* < 0.001; ORa = 1.314, *p* > 0.05) compared to females, but females are more likely to demonstrate a positive attitude (OR = 1.874, *p* < 0.001; ORa = 2.672, *p* < 0.01) and practice (OR = 1.401, *p* < 0.05; ORa = 1.921, *p* < 0.05). Age significantly impacts knowledge, attitude, and practices. Younger age groups, such as those aged 18–24 and 25–34, are more likely to have positive knowledge, attitudes, and practices compared to the reference group (55 and above) related to skin cancer, with strong associations observed (*p* < 0.001). Education levels are also linked to knowledge, attitude, and practices. Those with primary education or below show lower odds of having positive knowledge (OR = 0.718, *p* > 0.05; ORa = 0.735, *p* > 0.05) and practices (OR = 1.369, *p* < 0.05; ORa = 0.206, *p* > 0.05) but not attitudes. In contrast, those with higher secondary education exhibit higher odds of having positive knowledge (OR = 0.694, *p* < 0.001; ORa = 0.863, *p* > 0.05) and practices (OR = 1.052, *p* > 0.05; ORa = 0.387, *p* > 0.05) regarding skin cancer. Employment status significantly influences knowledge, attitude, and practices. Full-time employed individuals have higher odds of having positive knowledge (OR = 1.759, *p* < 0.001; ORa = 0.699, *p* > 0.05) and practices (OR = 0.986, *p* > 0.05; ORa = 2.1834, *p* > 0.05). Students exhibit higher odds of having positive knowledge (OR = 1.341, *p* < 0.05; ORa = 2.154, *p* < 0.05) and positive attitude (OR = 1.644, *p* < 0.01; ORa = 0.043, *p* < 0.05).

The determinants for the dependent variables (knowledge, attitude, and practice) are influenced by several demographic factors. For knowledge, factors such as gender, age, and education level play a significant role, with males, younger age groups (18–24 and 25–34), and higher education levels demonstrating increased odds. In terms of attitude, gender, age, and education also contribute, with males, younger age groups, and higher education levels exhibiting more positive attitudes. Regarding practice, gender, age, and education remain influential, with similar patterns observed. In addition, occupation is a determinant for practice, where full-time employed individuals show higher odds than other occupational categories. These findings emphasize the multifaceted nature of the determinants, showcasing the importance of tailoring interventions to address specific demographic characteristics to promote knowledge, positive attitudes, and desirable practices effectively.

#### 3.1.6. Source of Knowledge

**Figure 1:**
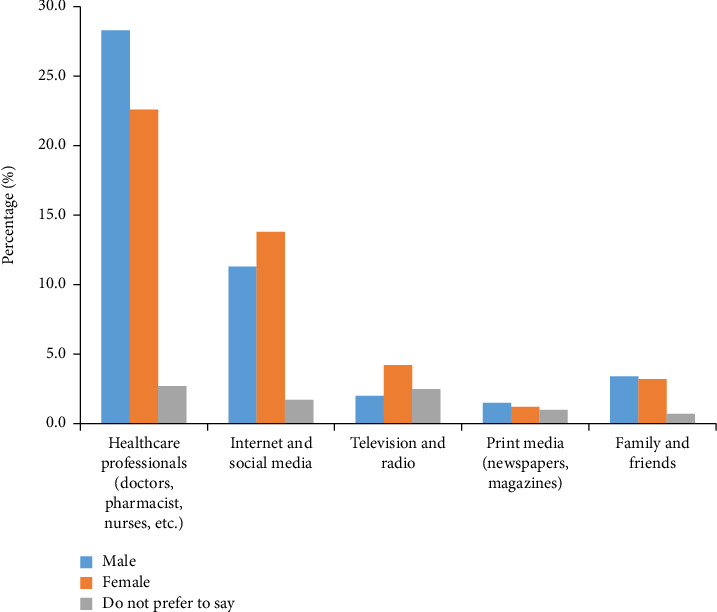
Source of knowledge among the general population.

Figure 1 provides valuable insights into the primary sources of knowledge about skin cancer within the general population. Healthcare professionals, such as doctors, pharmacists, and nurses, emerged as the most prominent sources of information, with 28.3% of males and 22.6% of females relying on them for skin cancer knowledge. Internet and social media also play a significant role, with 11.3% of males and 13.8% of females turning to online sources. Interestingly, television and radio have a smaller impact, with only 2.0% of males and 4.2% of females gaining knowledge from these mediums. These findings highlight the pivotal role of healthcare professionals and digital platforms in disseminating skin cancer information to the general population.

**Figure 2:**
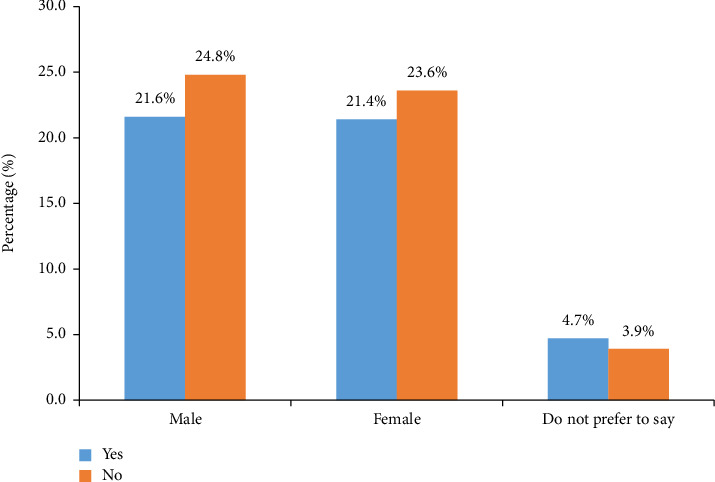
Have you encountered any knowledge campaigns or educational materials about skin cancer and its prevention?

The general population is exposed to skin cancer awareness campaigns and educational materials as shown in Figure 2. Approximately 21.6% of males and 21.4% of females have encountered knowledge campaigns, indicating a relatively balanced dissemination across genders. However, a significant portion, around 24.8% of males and 23.6% of females, have not come across these resources. These findings suggest that while a substantial portion of the population is exposed to skin cancer knowledge efforts, there remains an opportunity to expand the reach of these campaigns to ensure that a larger segment of the population is informed about skin cancer and its prevention.

### 3.2. Healthcare Professionals

#### 3.2.1. Demographic Characteristics

Table 6 provides a comprehensive overview of responses from healthcare professionals, categorized by their gender, age, and years of experience. Notably, most respondents were female (59.6%), with males comprising 37.0%. Physicians and nurses comprise the most significant groups among the respondents, with 19.6% and 18.7%, respectively. Among age groups, those aged 25–34 represent the largest segment (59.1%), and healthcare professionals with less than five years of experience are the most prevalent (66.5%). These data offer valuable insights into the demographics and experience levels of healthcare professionals included in the current study.

#### 3.2.2. Knowledge of Skin Cancer

Table 7 presents crucial insights into healthcare professionals' knowledge and knowledge regarding skin cancer. In response to questions about their knowledge of skin cancer and its causes, 38.3% of physicians, 11.3% of nurses, and 14.8% of pharmacists felt adequately knowledgeable. When asked about their ability to identify common signs and symptoms of skin cancer, 37.3% of physicians, 11.3% of nurses, and 12.6% of pharmacists responded affirmatively. Moreover, 23% of physicians, 3.9% of nurses, and 8.7% of pharmacists were aware of the latest guidelines and procedures for skin cancer screening and diagnosis. Notably, statistically significant variations were observed in responses across healthcare categories for the latter two questions (*p* value < 0.05).

#### 3.2.3. Attitude Toward Skin Cancer

Table 8 provides insights into healthcare professionals' attitudes toward skin cancer prevention and patient education. When asked about the importance of regular skin cancer screenings for at-risk patients, a significant majority viewed it as “very important,” with physicians (44%), nurses (13.0%), and pharmacists (14.8%) expressing the highest levels of importance. Conversely, when questioned about their confidence in educating patients about skin cancer prevention, the majority felt “very confident,” particularly physicians (20.8%), pharmacists (8.3%), and nurses (3%). Regarding the belief that healthcare providers play a crucial role in preventing skin cancer, a substantial proportion strongly agreed (31.7%), particularly physicians, pharmacists (11.7%), and nurses (13.5%). In comparison, a small minority strongly disagreed (3.8%), with others holding various viewpoints. These findings underscore the varying perspectives within the healthcare community.

#### 3.2.4. Practices of Skin Cancer Prevention and Management

Table 9 summarizes the responses from healthcare professionals regarding their practices and attitudes related to skin cancer education, screening, and their interest in further education. In the first question about patient education, approximately 23.9% of physicians, 4.8% of nurses, and 7.4% of pharmacists indicated that they always educate their patients about skin cancer and its prevention measures. For regular screening of at-risk patients for skin cancer, 27% of physicians, 3.9% of nurses, and 8.7% of pharmacists responded positively. Furthermore, when asked if they would be interested in receiving additional skin cancer education, 52.2% of physicians, 15.2% of nurses, and 19.6% of pharmacists expressed interest.

#### 3.2.5. Source of Knowledge

**Figure 3:**
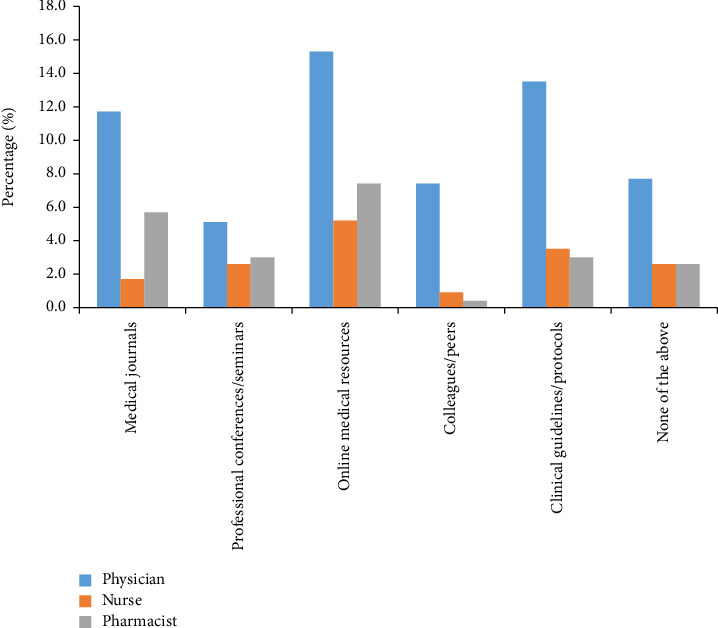
Source of knowledge among healthcare professionals.

Figure 3 illustrates the sources from which healthcare professionals gather information about skin cancer. Notably, medical journals are the most frequently consulted source, with 11.7% of physicians, 1.7% of nurses, and 5.7% of pharmacists turning to them. Professional conferences and seminars also play a significant role, with 5.1% of physicians, 2.6% of nurses, and 3.0% of pharmacists using them as a valuable source of information. Online medical resources are another prominent source, with 15.3% of physicians, 5.2% of nurses, and 7.4% of pharmacists using them as a source of information. Interestingly, clinical guidelines and protocols are particularly influential for physicians, with 13.5% citing them. Strikingly, the *p* value of < 0.001 indicates statistically significant variations in information sources across healthcare professionals.

## 4. Discussion

Skin cancer is an escalating health concern, ranking as one of the most prevalent cancer types worldwide [27]. Cancers, notably, stand as the second leading cause of mortality, following closely behind cardiovascular diseases [28]. To address this growing issue, the World Health Organization (WHO) has undertaken several initiatives to manage skin cancer [29]. These efforts include increasing public knowledge about skin cancer through health education, promoting more favorable attitudes, and enhancing preventive measures, as numerous risk factors have been identified [30]. Elevating societal consciousness regarding skin cancer has the potential to significantly curtail its high morbidity and mortality rates [31]. Education plays a pivotal role in increasing knowledge about the risks and symptoms of skin cancers and their early diagnosis and treatment [32]. The current study adds to a global understanding of skin cancer prevention by highlighting knowledge, attitudes, and practices in Pakistan, a LMIC.

### 4.1. General Population

The data collected regarding the level of knowledge revealed a direct correlation with educational attainment. Those with higher academic degrees exhibited a more profound understanding of skin cancer, a finding consistent with the research of Gizaw et al. [33]. Compared to studies conducted within specific populations, our study found that 38.1% of participants knew about the general risks associated with sunlight exposure and its connection to the development of skin cancer. In addition, 27.5% of participants reported having a family history of skin cancer as a significant risk factor for the disease. Nonetheless, in a study involving 387 Greek students, skin cancer (97.7%) was identified as the primary outcome, followed by skin aging in 72.9%. Similar results have also been observed among Arab and United States' students, indicating a consistent pattern of knowledge regarding the importance of skin cancer [34–36]. Regarding gender differences, it is noteworthy that men are less inclined toward skincare than women, which can be attributed to cultural gender roles [37]. This observation is reinforced by Almuqati et al.'s findings [35], where women displayed a higher level of knowledge regarding the consequences of sun exposure, encompassing aspects such as burns, hyperpigmentation, aging, and skin cancer. While the current study did not reveal significant differences in knowledge between genders, it was evident that female participants were more likely to adopt positive attitudes and practices concerning skincare and protection against the adverse effects of sun exposure and skin cancer. Regarding sources of information dissemination, the current study revealed that 53.6% of participants considered healthcare professionals, such as doctors, pharmacists, and nurses, as the primary source of information. This contrasts with the findings of Wan [38], who conducted a study in China's adult population and found that television advertisements were the predominant source of information, chosen by 52% of respondents for sun protection.

Conversely, another study conducted by Sandoval et al. in China indicated that only 8% of participants relied on professional sources such as doctors, dermatologists, and health campaigns for their information [11]. In our study, a greater proportion of participants relied on these authoritative sources, which can be attributed to the fact that in Pakistan, a LMIC, individuals typically trust healthcare professionals for guidance. Various protective measures have been recommended to reduce the incidence of skin cancer, with the current best practices being limiting exposure to sunlight, especially during peak hours, and using sunscreen [39]. In our study, 23.3% of participants sought shade during peak hours, and 29.7% used sunscreen as a protective measure to prevent skin cancer. These results are similar to the data provided by Almuqati et al. [35], who found that 23.6% of Arab students regularly use sunscreen. Our study further revealed that 29.0% of participants consistently used sunscreen, 37.6% used it occasionally, 18.7% rarely, and 14.8% never used it. These findings are consistent with a study on the Saudi population, which reported a regular sunscreen usage rate of 23.7% [24]. In terms of other sun protection measures, 55.4% of participants reported employing alternatives to sunscreen, with the use of caps and hats being the most common choice, selected by 32.1% of participants. These measures are also observed in Western populations across various regions, such as Mexico, where the use of sunglasses and caps for physical protection is prevalent among 73% and 66.9% of individuals who engage in beach sports [40]. Unfortunately, the current study findings indicate that substantial proportions of the Pakistani population exhibit suboptimal adherence to photoprotection measures, such as wearing sunscreen and seeking shade, and tend to engage in skin cancer risk behaviors, such as sunburn and tanning, a trend that aligns with studies conducted in the United States [41]. Moreover, the current study also highlights the need for public health programs that align with local needs. In countries with low skin cancer knowledge, such as Pakistan, these efforts should focus on educating through trusted sources, such as healthcare workers, on promoting protective behaviors. This approach can help address healthcare gaps in LMICs.

### 4.2. Healthcare Professionals

The lack of knowledge and clinical skills in diagnosing skin cancer has been documented in healthcare professionals of countries with high incidence rates as well [42–44]. Several reasons for these knowledge and practice gaps have been identified in these studies, including time constraints, inadequate training, and a lack of confidence. These findings closely parallel the results of our study. In our research, many healthcare professionals expressed a lack of confidence in educating patients about skin cancer prevention. Consequently, only 36.1% reported consistently advising or educating patients on skin cancer. However, more than half (64.4%) considered themselves knowledgeable about skin cancer and its causes, while 71.8% believed that screening was of paramount importance for at-risk patients. In contrast, a similar study conducted among Jordanian medical students yielded different results. A majority of the students (81%) demonstrated a solid understanding of skin cancer, and their attitudes toward skin cancer were generally positive [45]. The difference in self-perceived knowledge and attitudes toward skin cancer between healthcare professionals in our study and Jordanian medical students can be attributed to variations in education and training. Healthcare professionals who have already completed their education and training may have encountered limited exposure to comprehensive skin cancer education during their formal education. Consequently, their self-assessment of knowledge might be lower. In contrast, the Jordanian medical students currently undergoing medical education will likely benefit. The current study shows that medical journals emerge as the primary source of information for physicians, nurses, and pharmacists. Only a small minority, precisely 13.5% of physicians, turn to clinical guidelines and protocols as a source of information for skin cancer. In contrast, a study by Seetan et al. shows that 81% of medical students acquire knowledge from their medical school education [45]. This disparity can be attributed to medical students obtaining information from their academic institutions, while healthcare professionals rely more on medical journals. These results show a need for ongoing training programs to help healthcare professionals learn more about skin cancer and feel confident about discussing it with patients. In LMICs, such training can help healthcare workers better support skin cancer prevention efforts.

### 4.3. Importance of Artificial Intelligence (AI) Tools in the Early Diagnosis of Skin Cancer

To improve skin cancer detection and prevention, incorporating AI tools into clinical practice shows great potential. AI–based systems, particularly those focused on melanoma lesion classification [46], data preprocessing [47], and specialized detection architectures [48], can support healthcare professionals in early diagnosis, thereby improving patient outcomes, especially in underserved areas with limited access to specialized care. Future research should explore the integration of these technologies alongside public health initiatives for more effective skin cancer management.

### 4.4. Strengths and Limitations

This study on skin cancer in Pakistan exhibits several strengths that enhance its significance. First, its comprehensive approach, involving healthcare professionals and the general population, provides a better understanding of the prevailing knowledge, attitudes, and practices. The cross-sectional design captures a snapshot of the current scenario, aiding in immediate public health decision-making. The statistical analysis, considering gender, age, education, and employment status, offers a detailed exploration of variations in skin cancer knowledge. Ethical considerations, including approval from the institutional review board and a commitment to participant confidentiality, ensure the study's ethical integrity. These strengths collectively contribute to the reliability and relevance of the study's outcomes. Despite its strengths, this study has some inherent limitations. While providing valuable insights into a specific context, the regional focus on Lahore may limit the generalizability of the findings to the entire country. Reliance on self-reported data may introduce biases such as recall or social desirability bias, impacting the accuracy of responses. Despite these limitations, the study's findings contribute valuable insights into skin cancer in Pakistan.

## 5. Conclusion

Skin cancer ranks among the most prevalent cancers affecting both men and women. Notably, it is a highly preventable form of cancer. In conclusion, our cross-sectional study in Pakistan on the knowledge, attitude, and practices related to skin cancer among the general population and healthcare professionals reveals several significant findings. In the context of the general population, there is a noteworthy gender difference in knowledge, with males showing slightly higher knowledge levels and females demonstrating a more positive attitude and better preventive practices. Younger age groups exhibit more excellent knowledge, positive attitudes, and better practices regarding skin cancer. Education plays a role, with higher education levels associated with increased knowledge and practices. Overall, these results underscore the importance of targeted public health interventions in Pakistan to bridge gender and education gaps, enhance knowledge, and encourage proactive practices in the fight against skin cancer. This study highlights a need for further research in diverse populations and settings to develop adaptable public health strategies for skin cancer prevention, particularly in regions with limited healthcare access. In the context of healthcare professionals in Pakistan, the data reveal disparities in knowledge, with physicians displaying a higher level of knowledge than other healthcare categories. Significant proportions of healthcare providers acknowledge the importance of regular skin cancer screenings, and there is confidence in their ability to educate patients about prevention. However, a substantial portion still expresses the need for additional education, highlighting the ongoing demand for professional development in this field. Importantly, our findings underscore the necessity for targeted education initiatives, particularly for nurses, to bridge the knowledge gaps and enhance consistency in skin cancer prevention and management practices within the healthcare community. Addressing these disparities through focused training programs can significantly contribute to more effective skin cancer prevention and early detection strategies in the Pakistani healthcare system.

## Figures and Tables

**Table 1:** Demographic characteristics of the general population.

Demographics		Frequency	Percentage (%)
Gender	Male	189	46.4
	Female	183	45.0
	Do not prefer to say	35	8.6

Age	18–24	143	35.1
	25–34	147	36.1
	35–44	62	15.2
	45–54	29	7.1
	55 and above	26	6.4

Education	Primary education or below	30	7.4
	Secondary education	38	9.3
	Higher secondary education	55	13.5
	Bachelor's degree	187	45.9
	Master's degree or higher	97	23.8

Occupation	Employed (full-time)	106	26.0
	Employed (part-time)	55	13.5
	Self-employed	57	14.0
	Student	109	26.8
	Unemployed	63	15.5
	Retired	17	4.2

**Table 2:** Knowledge of skin cancer among the general population.

Question	Variables	Male (*n* = 183; 45%)	Female (*n* = 189; 46.4%)	Do not prefer to say (*n* = 35; 8.6%)	95% CI	*p* value
Have you ever heard of skin cancer?	Yes	148 (36.4%)	134 (32.9%)	12 (2.9%)	1.23–1.32	< 0.001
	No	41 (10.1%)	49 (12%)	23 (5.7%)		

How would you rate your knowledge about skin cancer?	Very knowledgeable	36 (8.8%)	26 (6.4%)	6 (1.5%)	2.06–2.20	0.066
	Moderately knowledgeable	102 (25.1%)	104 (25.6%)	12 (2.9%)		
	Not knowledgeable	51 (12.5%)	53 (13.0%)	17 (4.2%)		

Which of the following can prevent skin cancer?	Wearing sunscreen	68 (16.7%)	74 (18.2%)	5 (1.2%)	2.02–2.23	0.075
	Regular check-ups with a dermatologist	62 (15.2%)	53 (13.0%)	12 (2.9%)		
	Avoiding midday sun	33 (8.1%)	27 (6.6%)	8 (2.0%)		
	Do not know	26 (6.4%)	29 (7.1%)	10 (2.5%)		

Can you identify at least one common risk factor for skin cancer?	Excessive sun exposure	85 (20.9%)	66 (16.2%)	4 (1.0%)	2.17–2.43	0.002
	Family history of skin cancer	49 (12.0%)	52 (12.8%)	11 (2.7%)		
	Fair skin type	24 (5.9%)	24 (5.9%)	6 (1.5%)		
	Smoking	10 (2.5%)	16 (3.9%)	9 (2.2%)		
	Do not know	21 (5.2%)	25 (6.1%)	5 (1.2%)		

Which of the following signs or symptoms may indicate skin cancer?	New or changing moles	38 (9.3%)	26 (6.4%)	3 (0.7%)	2.59–2.86	0.032
	Unusual growth or lump on the skin	84 (20.6%)	72 (17.7%)	9 (2.2%)		
	Persistent itching or pain in a specific area	33 (8.1%)	37 (9.1%)	11 (2.7%)		
	Do not know	34 (8.4%)	48 (11.8%)	12 (2.9%)		

**Table 3:** Attitude toward skin cancer of the general population.

Question	Variables	Male (*n* = 183; 45%)	Female (*n* = 189; 46.4%)	Do not prefer to say (*n* = 35; 8.6%)	95% CI	*p* value
Do you believe that skin cancer is a significant health concern?	Yes	164 (40.3%)	148 (36.4%)	20 (4.9%)	1.15–1.22	< 0.001
	No	25 (6.1%)	35 (8.6%)	15 (3.7%)		

Are you concerned about the potential risks of skin cancer?	Yes	126 (31.0%)	124 (30.5%)	9 (2.2%)	1.32–1.41	< 0.001
	No	63 (15.5%)	59 (14.5%)	26 (6.4%)		

Do you think that skin cancer can be prevented by using sunscreen?	Yes	89 (21.9%)	78 (19.2%)	8 (2.0%)	1.87–2.05	0.078
	No	35 (8.6%)	29 (7.1%)	8 (2.0%)		
	Unsure	65 (16.0%)	76 (18.7%)	19 (4.7%)		

Do you believe it is important to have your skin checked regularly by a dermatologist is essential?	Yes	117 (28.7%)	86 (21.1%)	9 (2.2%)	1.68–1.85	< 0.001
	No	33 (8.1%)	38 (9.3%)	7 (1.7%)		
	Unsure	39 (9.6%)	59 (14.5%)	19 (4.7%)		

Which of the following preventive measures have you practiced to protect your skin from the sun?	Using sunscreen	57 (14.0%)	62 (15.2%)	2 (0.5%)	2.21–2.45	< 0.001
	Wearing protective clothing (e.g., hats and long sleeves)	71 (17.4%)	53 (13.0%)	7 (1.7%)		
	Seeking shade during peak sun hours	41 (10.1%)	38 (9.3%)	16 (3.9%)		
	Avoiding tanning beds or sunlamps	5 (1.2%)	10 (2.5%)	6 (1.5%)		
	None of the above	15 (3.7%)	20 (4.9%)	4 (1.0%)		

**Table 4:** Practices of skin cancer prevention and management among the general population.

Question	Variables	Male (*n* = 183; 45%)	Female (*n* = 189; 46.4%)	Do not prefer to say (*n* = 35; 8.6%)	95% CI	*p* value
Have you ever undergone a skin cancer screening or examination?	Yes	79 (19.4%)	59 (14.5%)	16 (3.9%)	1.57–1.67	0.099
	No	110 (27.0%)	124 (30.5%)	19 (4.7%)		

Have you ever performed a self-examination of your skin to check for signs of skin cancer?	Yes	86 (21.1%)	59 (14.5%)	7 (1.7%)	1.58–1.67	0.003
	No	103 (25.3%)	124 (30.5%)	28 (6.9%)		

How frequently do you use sun protection (sunscreen, protective clothing, etc.)?	Always	47 (11.5%)	65 (16.0%)	6 (1.5%)	2.09–2.29	0.160
	Sometimes	74 (18.2%)	64 (15.7%)	15 (3.7%)		
	Rarely	38 (9.3%)	32 (7.9%)	6 (1.5%)		
	Never	30 (7.4%)	22 (5.4%)	8 (2.0%)		

Have you ever had your skin checked by a doctor for signs of skin cancer?	Yes	67 (16.5%)	50 (12.3%)	8 (2.0%)	1.65–1.74	0.136
	No	122 (30.0%)	133 (32.7%)	27 (6.6%)		

If you notice any suspicious changes on your skin, what would be your next course of action?	Schedule an appointment with a healthcare professional	115 (28.3%)	98 (24.1%)	7 (1.7%)	1.54–1.68	< 0.001
	Monitor the changes without seeking medical advice	52 (12.8%)	55 (13.5%)	20 (4.9%)		
	Not sure	22 (5.4%)	30 (7.4%)	8 (2.0%)		

**Table 5:** Regression analysis.

Demographic	Knowledge		Attitude		Practice	
	OR	ORa	OR	ORa	OR	ORa
Gender						
Male	**1.413 (1.172-1.704) **⁣^∗∗∗^	1.314 (0.967–1.786)	**2.071 (1.563-2.743) **⁣^∗∗∗^	1.062 (0.780–1.446)	**1.600 (1.177-2.174) **⁣^∗∗^	1.390 (0.893–2.164)
Female	**1.327 (1.104-1.596) **⁣^∗∗^	1.582 (0.954–1.725)	**1.874 (1.419-2.474) **⁣^∗∗∗^	**2.672 (1.074-3.045) **⁣^∗∗^	**1.401 (1.030-1.905) **⁣^∗^	**1.921 (1.354-2.135) **⁣^∗^
Do not prefer to say	Reference					

Age						
18–24	**1.268 (1.019-1.576) **⁣^∗∗^	**2.980 (1.801-3.245) **⁣^∗∗∗^	**2.061 (1.483-2.862) **⁣^∗∗∗^	**5.356 (3.756-8.288) **⁣^∗∗∗^	1.102 (0.812–1.496)	**8.244 (7.652-9.090) **⁣^∗∗∗^
25–34	**1.560 (1.238-1.967) **⁣^∗∗∗^	**4.717 (3.245- 5.304) **⁣^∗∗∗^	**2.533 (1.807-3.550) **⁣^∗∗∗^	**4.519 (4.186-5.145) **⁣^∗∗∗^	1.215 (0.897–1.646)	**2.245 (1.245-3.651) **⁣^∗∗∗^
35–44	1.072 (0.849–1.353)	**4.145 (3.315-5.541) **⁣^∗^	1.328 (0.941–1.875)	**3.214 (1.346-5.451) **⁣^∗^	1.027 (0.734–1.436)	**4.062 (1.076-5.245) **⁣^∗^
45–54	1.009 (0.773–1.317)	1.050 (0.195–5.648)	1.248 (0.840–1.852)	1.032 (0.192–5.552)	0.848 (0.564–1.275)	2.007 (0.512–3.145)
55 and above	Reference					

Education						
Primary education or below	0.718 (0.565–0.914)	0.735 (0.115–4.691)	**0.483 (0.351-0.665) **⁣^∗∗∗^	0.638 (0.102–3.975)	**1.369 (1.045-1.794) **⁣^∗^	0.206 (0.032–1.337)
Secondary education	0.802 (0.634–1.015)	0.370 (0.069–1.993)	**0.510 (0.380-0.684) **⁣^∗∗∗^	0.344 (0.064–1.854)	1.049 (0.807–1.362)	0.232 (0.047–1.157)
Higher secondary education	**0.694 (0.567-0.850) **⁣^∗∗∗^	0.863 (0.161–4.610)	**0.500 (0.384-0.652) **⁣^∗∗∗^	0.779 (0.147–4.136)	1.052 (0.835–1.325)	0.387 (0.077–1.938)
Bachelor's degree	**0.811 (0.685-0.960) **⁣^∗^	0.687 (0.172–2.734)	0.848 (0.693–1.037)	0.630 (0.163–2.438)	0.989 (0.830–1.177)	**0.258 (0.069-0.966) **⁣^∗^
Master's degree or higher	Reference					

Occupation						
Employed (full-time)	**1.759 (1.337-2.314) **⁣^∗∗∗^	0.699 (0.092–5.342)	**2.198 (1.493-3.236) **⁣^∗∗∗^	0.997 (0.137–7.248)	0.986 (0.696–1.396)	2.183 (1.514–3.145)
Employed (part-time)	**1.682 (1.248-2.267) **⁣^∗^	0.173 (0.023–1.297)	1.201 (0.813–1.776)	0.234 (0.032–1.693)	1.126 (0.781–1.624)	2.263 (0.268–19.121)
Self-employed	**1.465 (1.107-1.940) **⁣^∗∗^	**0.063 (0.009-0.417) **⁣^∗^	1.304 (0.883–1.928)	**0.073 (0.011-0.478) **⁣^∗∗^	0.877 (0.604–1.274)	0.741 (0.097–5.648)
Unemployed	1.225 (0.941–1.594)	**0.134 (0.019-0.949) **⁣^∗^	**1.524 (1.031-2.251) **⁣^∗^	**0.124 (0.018-0.856) **⁣^∗^	0.704 (0.481–1.032)	1.214 (0.958–1.554)
Student	**1.341 (1.041-1.729) **⁣^∗^	**2.154 (1.245-2.245) **⁣^∗^	**1.644 (1.131-2.389) **⁣^∗∗^	**0.043 (0.006-0.307) **⁣^∗^	0.898 (0.633–1.273)	1.584 (0.202–12.440)
Retired	Reference					

*Note:* Bold values represent statistically significant results.

⁣^∗^* p* < 0.05.

⁣^∗∗^* p* < 0.01.

⁣^∗∗∗^* p* < 0.001.

**Table 6:** Demographic characteristics of healthcare professionals.

Demographics		Physician	Nurse	Pharmacist	Total
Gender	Male	67 (29.1%)	1 (0.4%)	17 (7.4%)	85 (37.0%)
	Female	69 (30%)	34 (14.8%)	34 (14.8%)	137 (59.6%)
	Do not prefer to say	5 (2.1%)	3 (1.3%)	0 (0%)	8 (3.5%)

Age	18–24	22 (9.6%)	5 (2.2%)	18 (7.8%)	45 (19.6%)
	25–34	76 (37.8%)	21 (9.1%)	28 (12.2%)	136 (59.1%)
	35–44	24 (10.4%)	10 (4.3%)	4 (1.7%)	38 (16.5%)
	45–54	5 (2.1%)	2 (0.9%)	1 (0.4%)	8 (3.5%)
	55 and above	3 (1.3%)	0 (0%)	0 (0%)	3 (1.3%)

Experience	Less than 5 years	90 (39.18%)	20 (8.7%)	43 (18.7%)	153 (66.5%)
	5–10 years	24 (10.4%)	12 (5.2%)	6 (2.6%)	42 (18.3%)
	More than 10 years	27 (11.7%)	6 (2.6%)	2 (0.9%)	35 (15.2%)

**Table 7:** Knowledge of skin cancer among healthcare professionals.

Question	Variables	Physician (*n* = 141; 61.3%)	Nurse (*n* = 38; 16.5%)	Pharmacist (*n* = 51; 22.2%)	95% CI	*p* value
Do you feel adequately knowledgeable about skin cancer and its causes?	Yes	88 (38.3%)	26 (11.3%)	34 (14.8%)	1.49–1.71	0.192
	No	17 (7.4%)	3 (1.3%)	7 (3.0%)		
	Unsure	36 (15.7%)	9 (3.9%)	10 (4.3%)		

Can you identify common signs and symptoms of skin cancer in a patient?	Yes	86 (37.3%)	26 (11.3%)	29 (12.6%)	1.49–1.71	< 0.001
	No	23 (9.9%)	1 (0.4%)	16 (7.0%)		
	Unsure	32 (13.9%)	11 (4.8%)	6 (2.6%)		

Are you aware of the latest guidelines and procedures for skin cancer screening and diagnosis?	Yes	53 (23.0%)	9 (3.9%)	20 (8.7%)	1.84–2.05	< 0.001
	No	51 (22.2%)	5 (2.2%)	22 (9.6%)		
	Unsure	37 (16.0%)	24 (10.4%)	9 (3.9%)		

**Table 8:** Attitude toward skin cancer among healthcare professionals.

Question	Variables	Physician (*n* = 141; 61.3%)	Nurse (*n* = 38; 16.5%)	Pharmacist (*n* = 51; 22.2%)	95% CI	*p* value
How important are regular skin cancer screenings for at-risk patients?	Very important	101 (44.0%)	30 (13.0%)	34 (14.8%)	1.30–1.48	0.007
	Somewhat important	25 (10.8%)	5 (2.2%)	10 (4.3%)		
	Not important	15 (6.5%)	3 (1.3%)	7 (3.0%)		

How confident do you feel in educating patients about skin cancer prevention?	Very confident	48 (20.8%)	7 (3.0%)	19 (8.3%)	1.75–1.93	0.001
	Somewhat confident	69 (30.0%)	25 (10.9%)	25 (10.9%)		
	Not confident	24 (10.4%)	6 (2.6%)	7 (3.0%)		

Do you believe healthcare providers have a crucial role in preventing skin cancer?	Strongly agree	73 (31.7%)	31 (13.5%)	27 (11.7%)	1.65–1.95	< 0.001
	Agree	31 (13.4%)	3 (1.3%)	13 (5.7%)		
	Neutral	20 (8.7%)	2 (0.9%)	6 (11.8%)		
	Disagree	9 (4.0%)	2 (0.9%)	4 (1.7%)		
	Strongly disagree	8 (3.4%)	0 (0%)	1 (0.4%)		

**Table 9:** Practices of skin cancer prevention and management among healthcare professionals.

Question	Variables	Physician (*n* = 141; 61.3%)	Nurse (*n* = 38; 16.5%)	Pharmacist (*n* = 51; 22.2%)	95% CI	*p* value
How often do you educate your patients about skin cancer and its prevention measures?	Always	55 (23.9%)	11 (4.8%)	17 (7.4%)	1.90–2.15	0.239
	Occasionally	48 (20.9%)	16 (7.0%)	17 (7.4%)		
	Rarely	27 (11.7%)	9 (3.9%)	8 (3.5%)		
	Never	11 (4.8%)	2 (0.9%)	9 (3.9%)		

Do you regularly screen at-risk patients for signs of skin cancer?	Yes	62 (27.0%)	9 (3.9%)	20 (8.7%)	1.54–1.67	0.071
	No	79 (34.3%)	29 (12.6%)	31 (13.5%)		

Would you be interested in receiving additional skin cancer education, if available?	Yes	120 (52.2%)	35 (15.2%)	45 (19.6%)	1.09–1.17	0.064
	No	21 (9.1%)	3 (1.3%)	6 (2.6%)		

## Data Availability

The data that support the findings of this study are available from the corresponding author upon reasonable request.

## Supporting Information

Supporting 1. Questionnaire 1 and 2.
