# Supplementary material for: A Study of Skin Cancer Knowledge, Attitudes, and Preventive Practices Among Healthcare Professionals and the General Population in Pakistan: Insights for Healthcare Interventions and Policy Development
Source: J Skin Cancer. 2025 Mar 20;2025:3035274. doi: 10.1155/jskc/3035274 (PMC11949603; doi:10.1155/jskc/3035274)
Supplement: Supporting Information — Additional supporting information can be found online in the Supporting Information section. [file 3035274.f1.docx]

# **Knowledge, Attitude, and Practice (KAP) survey of skin cancer among general population in Pakistan (Questionnaire 1)**

# **Section 1: Demographic Information**

1. Gender:
2. Male
3. Female
4. Don't prefer to say
5. Age:
6. 18-24
7. 25-34
8. 35-44
9. 45-54
10. 55 and above
11. Education:
12. Primary education or below
13. Secondary education
14. Higher secondary education
15. Bachelor's degree
16. Master's degree or higher
17. Occupation:
18. Employed (full-time)
19. Employed (part-time)
20. Self-employed
21. Unemployed
22. Student
23. Retired

# **Section 2: Knowledge about Skin Cancer**

1. Have you ever heard of skin cancer?
2. Yes
3. No
4. How would you rate your knowledge about skin cancer?
5. Very knowledgeable
6. Moderately knowledgeable
7. Not knowledgeable
8. Which of the following can prevent skin cancer?
9. Wearing sunscreen
10. Regular check-ups with a dermatologist
11. Avoiding midday sun
12. Don't know
13. Can you identify at least one common risk factor for skin cancer?
    1. Excessive sun exposure
    2. Family history of skin cancer
    3. Fair skin type
    4. Smoking
    5. Don't know
14. Which of the following signs or symptoms may indicate skin cancer?
    1. New or changing moles
    2. Unusual growth or lump on the skin
    3. Persistent itching or pain in a specific area
    4. Don't know

# **Section 3: Attitudes Toward Skin Cancer**

1. Do you believe that skin cancer is a significant health concern?
2. Yes
3. No
4. Are you concerned about the potential risks of skin cancer?
5. Yes
6. No
7. Do you think that skin cancer can be prevented by using sunscreen?
8. Yes
9. No
10. Unsure
11. Do you believe it's important to have your skin checked regularly by a dermatologist is essential?
12. Yes
13. No
14. Unsure
15. Which of the following preventive measures have you practiced to protect your skin from the sun?
16. Using sunscreen
17. Wearing protective clothing (e.g., hats, long sleeves)
18. Seeking shade during peak sun hours
19. Avoiding tanning beds or sunlamps
20. None of the above

# **Section 4: Practices Related to Skin Cancer**

1. Have you ever undergone a skin cancer screening or examination?
2. Yes
3. No
4. Have you ever performed a self-examination of your skin to check for signs of skin cancer?
5. Yes
6. No
7. How frequently do you use sun protection (sunscreen, protective clothing, etc.)?
8. Always
9. Sometimes
10. Rarely
11. Never
12. Have you ever had your skin checked by a doctor for signs of skin cancer?
13. Yes
14. No
15. If you notice any suspicious changes on your skin, what would be your next course of action?
16. Schedule an appointment with a healthcare professional
17. Monitor the changes without seeking medical advice
18. Not sure

# **Section 5: Information Sources and Awareness Campaigns**

1. Where do you usually seek information about health-related issues?
   1. Healthcare professionals (doctors, pharmacists, nurses, etc.)
   2. Internet and social media
   3. Television and radio
   4. Print media (newspapers, magazines)
   5. Family and friends
2. Have you encountered any awareness campaigns or educational materials about skin cancer and its prevention?
3. Yes
4. No

# **Knowledge, Attitude, and Practice (KAP) Survey of skin cancer among healthcare professionals in Pakistan (Questionnaire 2)**

# **Section 1: Demographic Information**

1. Gender:
   1. Male
   2. Female
   3. Don't prefer to say
2. Age:
3. 18-24
4. 25-34
5. 35-44
6. 45-54
7. 55 and above
8. Professional designation:
9. Physician
10. Nurse
11. Pharmacist
12. Experience:
13. Less than 5 years
14. 5-10 years
15. More than 10 years

# **Section 2: Knowledge about Skin Cancer**

1. Do you feel adequately knowledgeable about skin cancer and its causes?
2. Yes
3. No
4. Unsure
5. Can you identify common signs and symptoms of skin cancer in a patient?
6. Yes
7. No
8. Unsure
9. Are you aware of the latest guidelines and procedures for skin cancer screening and diagnosis?
10. Yes
11. No
12. Unsure

# **Section 3: Attitudes Toward Skin Cancer**

1. How important are regular skin cancer screenings for at-risk patients?
2. Very important
3. Somewhat important
4. Not important
5. How confident do you feel in educating patients about skin cancer prevention?
6. Very confident
7. Somewhat confident
8. Not confident
9. Do you believe healthcare providers have a crucial role in preventing skin cancer?
10. Strongly agree
11. Agree
12. Neutral
13. Disagree
14. Strongly disagree

# **Section 4: Practices Related to Skin Cancer**

1. How often do you educate your patients about skin cancer and its prevention measures?
2. Always
3. Occasionally
4. Rarely
5. Never
6. Do you regularly screen at-risk patients for signs of skin cancer?
7. Yes
8. No
9. Would you be interested in receiving additional skin cancer education, if available?
10. Yes
11. No

# **Section 5: Sources of Information**

1. Where do you usually acquire information about skin cancer?
2. Medical journals
3. Professional conferences/seminars
4. Online medical resources
5. Colleagues/peers
6. Clinical guidelines/protocols
7. None of the above
